# Biochar-biostimulant combinations enhance nodulation, yield, and antioxidant defense in mungbean under reduced NPK fertilization

**DOI:** 10.1038/s41598-026-63172-0

**Published:** 2026-07-30

**Authors:** Mehedi Amin, Md. Nasir Hossain Sani, Md. Mahbubur Rahman, Suraiya Akter, Md. Riaz Mahabub, Sayed Mohammad Mohsin, Mohammed Ariful Islam, Jean Wan Hong Yong, Abu Noman Faruq Ahmmed

**Affiliations:** 1 Department of Plant Pathology, Sher-e-Bangla Agricultural University, Sher-e-Bangla Nagar, Dhaka, 1207 Bangladesh; 2 Department of Nutrition and Food Engineering, Daffodil International University, Savar, Dhaka, 1216 Bangladesh; 3 Department of Biosystems and Technology, Swedish University of Agricultural Sciences (SLU), P.O. Box 190, 234 22 Lomma, Sweden; 4 Brightlands Future Farming Institute, Faculty of Science and Engineering, Maastricht University, 5928 SX Venlo, The Netherlands; 5 Department of Agricultural Chemistry, Sher-e-Bangla Agricultural University, Sher-e- Bangla Nagar, Dhaka, 1207 Bangladesh

**Keywords:** Antioxidants, Humic acid, Plant-microbe interactions, Rhizobacteria, Oxidative stress, Soil health, Biotechnology, Ecology, Ecology, Environmental sciences, Microbiology, Plant sciences

## Abstract

Reducing mineral fertilizer inputs while maintaining crop productivity is a central challenge for sustainable grain legume production. This study evaluated whether biochar–biostimulant combinations could improve mungbean (*Vigna radiata* L.) performance under reduced NPK fertilization. A polytunnel pot experiment was conducted using a completely randomized 2 × 4 factorial design with two NPK levels, 100% and 50% of the recommended dose, and four treatments: biochar alone, biochar + humic acid, biochar + *Bacillus amyloliquefaciens*, and biochar + *Pseudomonas fluorescens*. Biostimulant addition to biochar improved plant growth, pigment status, and antioxidant response, with the strongest responses observed under reduced NPK supply. Under 50% NPK, biochar + *P. fluorescens* increased grain yield by approximately 90% compared with biochar alone and maintained grain yield (4.00 g plant⁻¹) comparable to the 100% NPK biochar-only control (3.85 g plant⁻¹). It also enhanced nodulation, increased total chlorophyll by approximately 60%, and reduced malondialdehyde by approximately 50%. Grain weight was positively associated with nodule number and ascorbic acid content and negatively associated with H₂O₂ content. These findings support biochar-assisted *P. fluorescens* supplementation as a promising strategy for sustaining mungbean productivity under reduced NPK input, warranting further field validation for broader applicability.

**Supplementary Information:**

The online version contains supplementary material available at 10.1038/s41598-026-63172-0.

## Introduction

Global food demand continues to increase, while the sustainability of intensive crop production is increasingly constrained by high dependence on synthetic fertilizers. Although mineral fertilizers have substantially improved crop productivity, their excessive or inefficient use can contribute to nutrient leaching, eutrophication, soil acidification, greenhouse gas emissions, and deterioration of soil biological functioning^1–5^. These concerns highlight the need for nutrient-management strategies that maintain crop productivity while reducing fertilizer inputs and associated environmental impacts^6,7^.

Bio-based soil amendments and plant biostimulants offer complementary opportunities to improve resource-use efficiency in crop production. Organic nutrient sources, including composts, digestates, and other recycled amendments, can improve soil structure, increase organic matter inputs, and contribute to circular nutrient management^8–11^. However, nutrient release from organic amendments may be slower or less synchronized with crop demand than that from mineral fertilizers, which can limit plant performance during periods of high nutrient requirement^12–14^. Under such conditions, plants may experience nutrient limitation, reduced photosynthetic performance, and oxidative imbalance reflecting an increased generation of reactive oxygen species (ROS) relative to antioxidant capacity^15,16^. Integrated approaches are therefore needed to support nutrient acquisition, rhizosphere functioning, and stress tolerance when mineral fertilizer inputs are reduced.

Biochar and biostimulants have attracted increasing interest as components of such integrated systems^17,18^. Biochar, a carbon-rich material produced through biomass pyrolysis, can improve selected soil physical and chemical properties, including water retention, nutrient retention, and cation exchange capacity, although its effects depend strongly on feedstock, pyrolysis conditions, soil type, and application rate^19,20^. Plant biostimulants, including humic substances (HS) and plant growth-promoting rhizobacteria (PGPR), have been reported to enhance plant performance through effects on root development, nutrient acquisition, phytohormone-related processes, and stress regulation^21–23^. Humic substances have been associated with improved root architecture and nutrient uptake^24,25^, acting primarily as non-microbial amendments that condition the rhizosphere and enhance nutrient availability. PGPR, by contrast, act through direct microbial mechanisms: *Bacillus amyloliquefaciens* is widely recognized for its spore-forming biofertilization and biocontrol activity, while *Pseudomonas fluorescens* is a rhizosphere-competent species associated with siderophore-mediated nutrient acquisition and induced systemic tolerance to stress^26–28^. Together, these biostimulants represent complementary non-microbial and microbial approaches for improving nutrient acquisition and physiological performance under reduced fertilizer input.

Combining biochar with biostimulants may further enhance these effects by improving rhizosphere conditions and supporting microbial activity^29^. Biochar can provide a porous carrier matrix and protective microhabitats that may facilitate microbial establishment, nutrient retention, and root–microbe interactions^30^. In parallel, HS may influence nutrient exchange, root signaling, and microbial activity in the rhizosphere, potentially complementing PGPR-mediated functions^31^. Previous studies have reported beneficial effects of biochar–microbe or biochar–humic combinations on soil enzyme activity, nutrient availability, antioxidant metabolism, and plant growth in different cropping systems^32^. However, much of this evidence derives from cereal or vegetable systems evaluated under a single fertilization regime, and comparative evidence on how contrasting biostimulant types perform in combination with biochar under reduced mineral fertilization remains limited, particularly in grain legumes where nodulation offers an additional, biologically distinct route to compensate for reduced nutrient supply.

This gap is particularly relevant for mungbean (*Vigna radiata* L.), a short-duration grain legume cultivated across South and Southeast Asia whose productivity depends on both external nutrient supply and effective nodulation for biological nitrogen fixation. Because nodulation represents the principal mechanism by which mungbean can offset reduced mineral nitrogen availability, it offers a direct physiological indicator of whether biostimulant supplementation can functionally compensate for reduced NPK input. In addition, reduced nutrient availability can affect photosynthetic pigment formation and increase oxidative pressure, making pigment status and antioxidant-related traits useful indicators of physiological resilience. Assessing nodulation, photosynthetic pigments, oxidative-stress markers, and antioxidant responses together can therefore provide a more integrated understanding of how biochar–biostimulant combinations influence yield formation under reduced NPK fertilization. Moreover, limited information is available on how humic acid and PGPR-based biostimulant supplementation under biochar-amended conditions differentially affects nodulation, physiological performance, oxidative-stress regulation, and yield formation under reduced NPK input.

Therefore, this study evaluated the effects of humic acid, *Bacillus amyloliquefaciens*, or *Pseudomonas fluorescens* on mungbean growth, physiological traits, antioxidant responses, nodulation, and yield under full and reduced NPK fertilization in a biochar-amended system. We hypothesized that biochar–biostimulant combinations would improve mungbean performance under reduced NPK input compared with biochar alone, and that these improvements would be associated with enhanced nodulation, pigment accumulation, antioxidant defense, and yield-related traits. The findings are intended to contribute to the development of more sustainable nutrient-management strategies for legume production.

## Materials and methods

### Plant material, experimental site and growth condition

Seeds of mungbean (*Vigna radiata* L.) cultivar BARI Mung-6 were obtained from the Bangladesh Agricultural Research Institute (BARI), Joydebpur, Bangladesh. Uniform, disease-free seeds were surface sterilized with 70% ethanol, rinsed thoroughly with distilled water, and soaked overnight in distilled water under dark conditions to promote uniform germination. The experiment was conducted inside a polytunnel at Sher-e-Bangla Agricultural University, Bangladesh, from February to May 2025. During the experimental period, the average daytime temperature inside the polytunnel was 28 ± 3 °C, the average night-time temperature was 20 ± 2 °C, and relative humidity ranged from 60% to 75%, measured daily at 09:00 and 15:00. A pot experiment was used to allow controlled comparison of amendment effects under relatively uniform soil and environmental conditions. Each 4-L plastic pot received 3.0 kg of air-dried soil mixed with inert sand at 30% (v/v) to improve drainage and maintain a uniform growth medium. Biochar was incorporated into the potting mixture at 5% (v/v), a rate selected to provide sufficient amendment volume for evaluating biostimulant responses under biochar-amended conditions while maintaining suitable potting-medium structure. Pots were arranged in a completely randomized design within the polytunnel and were periodically re-randomized during the experiment to minimize positional effects. Irrigation was applied uniformly to all pots as required to maintain adequate soil moisture while avoiding waterlogging. The physicochemical properties of the experimental soil and biochar are presented in Table 1.

**Table 1:** Physio**-**chemical characterization of biochar and soil.

Component	Biochar	Soil
pH	6.9	6.3
EC (mS cm^−1^)	2.24	0.30
OM (%)	–	2.2
Bulk density (kg L^−1^)	0.28	1.22
Total N (%)	0.63	0.14
Total C (%)	68	2.1
P (mg kg^−1^)	321	11.2
Total K (mg kg^− 1^)	2378	23.4
Surface area (m² g⁻¹)	182	–
Pore volume (cm³ g⁻¹)	0.31	–
Ash content (%)	12.4	–

### Biochar, rhizobacteria, humic acid: sources, characterization and formulations

The biochar used in this study was produced from mixed timber residues by slow pyrolysis at 500–550 °C under oxygen-limited conditions and was obtained from Green Loop Ltd., Bangladesh. Its physicochemical properties included pH, electrical conductivity, bulk density, total C, total N, total P, total K, surface area, pore volume, and ash content (Table 1). Biochar was applied on a volume basis rather than a weight basis.

Commercial humic acid (Sigma–Aldrich, USA; ≥85% humic substances) was used as a non-microbial biostimulant. A stock solution was prepared by dissolving the powdered material in deionized water under continuous magnetic stirring at room temperature until complete solubilization. Two plant growth-promoting rhizobacterial (PGPR) strains were obtained from the SAU Plant Pathology Laboratory culture collection: *Bacillus amyloliquefaciens* BAM-5, isolated from the mungbean rhizosphere at the Sher-e-Bangla Agricultural University farm, Dhaka, and *Pseudomonas fluorescens* PF-12, isolated from the potato rhizosphere in Munshiganj, Bangladesh. Both strains were identified using standard morphological and biochemical tests following *Bergey’s Manual of Systematic Bacteriology* and were screened for plant growth-promoting traits, including indole-3-acetic acid production, phosphate solubilization, siderophore production, ACC deaminase activity, and growth on N-free medium (Table 2). The strains were maintained as glycerol stocks (20%, v/v) at −80 °C, cultured in nutrient broth at 30 °C for 48 h with shaking at 150 rpm, freeze-dried using 10% skim milk as a cryoprotectant, and reconstituted in sterile distilled water immediately before application. Final inoculum densities were adjusted to approximately 1 × 10⁸ CFU mL⁻¹ for BAM-5 and 1 × 10⁹ CFU mL⁻¹ for PF-12. These strain-specific inoculum densities were selected based on reported effective PGPR ranges for legume inoculation and the previously observed growth characteristics of each strain^33,34^. Although the inoculants were selected based on their characterized plant growth-promoting traits, root colonization, rhizosphere population dynamics, and persistence of the introduced strains were not directly quantified during the experiment.

**Table 2:** Biochemical identification and functional traits of PGPR strains.

Parameter	Bacillus amyloliquefaciens BAM-5	Pseudomonas fluorescens PF-12
Gram stain	Positive rod	Negative rod
Endospore formation	+	−
Oxidase	−	+
Catalase	+	+
Fluorescent pigment (King’s B)	−	+
Gelatin hydrolysis	+	+
Starch hydrolysis	+	−
Arginine dihydrolase	ND	+
Growth at 4 °C	−	+
Growth at 41 °C	ND	−
IAA production (µg mL⁻¹)	34.2 ± 2.1	41.7 ± 3.0
Phosphate solubilization (µg mL⁻¹ P)	28.6 ± 1.8	35.2 ± 2.2
Siderophore production (CAS halo, mm)	5 ± 0.5	8 ± 0.7
ACC deaminase activity (µmol α-KB mg⁻¹ h⁻¹)	2.4 ± 0.2	3.1 ± 0.3
Nitrogen fixation (N-free medium)	+	+

*Values are mean ± SD (*n* = 3). + = positive; − = negative; ND = not determined. IAA = indole-3-acetic acid; CAS = chrome azurol S; ACC = 1-aminocyclopropane-1-carboxylate; α-KB = α-ketobutyrate. *.

### Experimental setup and treatments

The experiment was conducted as a 2 × 4 factorial arrangement in a completely randomized design (CRD) with five replications. The two experimental factors were: (i) NPK fertilization level, comprising 100% or 50% of the recommended dose, and (ii) biostimulant treatment, comprising biochar alone, biochar + humic acid, biochar + *B. amyloliquefaciens* BAM-5, and biochar + *P. fluorescens* PF-12. Biochar was included as the common amendment background in all treatment combinations, and the biochar-only treatment served as the control within each NPK level. The recommended fertilizer dose, following Hossain et al.^35^, was 20 kg ha⁻¹ urea, 50 kg ha⁻¹ triple superphosphate (TSP), and 45 kg ha⁻¹ muriate of potash (MOP). These field rates were proportionally converted to a per-pot basis according to the amount of soil used in each pot. TSP and MOP were applied as basal doses during pot preparation, whereas urea was split-applied, with 50% applied at sowing and 50% at 20 days after sowing (DAS).

Biochar was incorporated at 5% (v/v) by thoroughly mixing it with the growth medium one week before sowing to allow equilibration within the pot matrix. This rate was selected for controlled pot evaluation based on previous pot-based biochar studies and was intended to provide sufficient amendment volume for assessing biochar–biostimulant interactions without being interpreted as a direct field-rate recommendation^36,37^. Based on the measured biochar bulk density of 0.28 kg L⁻¹, the 5% (v/v) pot application rate corresponds approximately to 14 t ha⁻¹ when a shallow 10 cm field incorporation depth is assumed. Humic acid and PGPR were applied as soil drenches at sowing and again at 20 DAS. The humic acid treatment consisted of a 2% (w/v) aqueous solution. The PGPR suspensions were prepared immediately before use from freeze-dried powders to achieve the target viable cell concentrations described above. Each pot received a total of 500 mL PGPR suspension during the experiment, applied as two equal soil drenches of 250 mL pot⁻¹ at sowing and 20 DAS. Based on the adjusted inoculum densities, this corresponded to approximately 2.5 × 10¹⁰ CFU pot⁻¹ per application for *Bacillus amyloliquefaciens* BAM-5 and 2.5 × 10¹¹ CFU pot⁻¹ per application for *Pseudomonas fluorescens* PF-12. All treatment combinations, including the biochar-only control at both NPK levels, are presented in Table 3.

**Table 3:** Summary of experimental treatments, formulations and application rates.

Treatment	NPK dose	Biochar	Biostimulant type	Formulation and concentration
T0	100% NPK	5% (v/v)	None (Control)	-
	50% NPK	5% (v/v)	None (Control)	-
T1	100% NPK	5% (v/v)	Humic acid (HA)	2% (w/v) solution
	50% NPK	5% (v/v)	Humic acid (HA)	2% (w/v) solution
T2	100% NPK	5% (v/v)	PGPR (*B. amyloliquefaciens*)	2 g powder in 500 mL water
	50% NPK	5% (v/v)	PGPR (*B. amyloliquefaciens*)	2 g powder in 500 mL water
T3	100% NPK	5% (v/v)	PGPR (*P. fluorescens*)	3 g powder in 500 mL water
	50% NPK	5% (v/v)	PGPR (*P. fluorescens*)	3 g powder in 500 mL water

T0–T3 were applied under both 100% and 50% NPK levels. T0 represents the biochar-only control with no additional biostimulant; T1, T2, and T3 represent biochar combined with humic acid, *Bacillus amyloliquefaciens* BAM-5, and *Pseudomonas fluorescens* PF-12, respectively. Biochar was incorporated at 5% (v/v) in all treatments. PGPR, plant growth-promoting rhizobacteria.

### Growth, yield, and biomass traits

Growth traits, including plant height, number of primary branches, and total leaf area, were recorded at the flowering stage to represent peak vegetative development. Root-related traits were measured only at final harvest to avoid disturbing plant growth during the experiment. At harvest, plants were carefully uprooted to record root length and the number of visible healthy nodules per plant. Yield-related traits were assessed at maturity by recording the number of pods per plant, number of grains per plant, and grain weight per plant. For biomass determination, each harvested plant was separated into shoots and roots, and fresh weight (FW) was recorded immediately. The samples were then oven-dried at 70 °C for 48 h to determine dry weight (DW).

### Physiological and biochemical analyses

Relative water content (RWC), proline content, and photosynthetic pigment concentrations were determined using fully expanded leaf samples collected from each treatment. For RWC, fresh weight (FW) was recorded immediately after sampling. The leaf blades were then soaked in distilled water for 24 h in the dark to obtain turgid weight (TW), followed by oven drying at 80 °C for 72 h to determine dry weight (DW). RWC was calculated according to Barrs and Weatherley^38^ as:

$${\text{RWC }}\left( \% \right){\text{ }} = {\text{ }}\left[ {\left( {{\mathrm{FW}}\, - \,{\mathrm{DW}}} \right){\text{ }}/{\text{ }}\left( {{\mathrm{TW}}\, - \,{\mathrm{DW}}} \right)} \right]{\text{ }} \times {\text{ 1}}00$$

For proline determination, 0.5 g of fresh leaf tissue was homogenized in 3% sulfosalicylic acid and centrifuged at 11,500 ×g for 15 min. One milliliter of the supernatant was mixed with acid ninhydrin and glacial acetic acid, incubated at 100 °C for 1 h, cooled, and extracted with 4 mL toluene. The absorbance of the upper phase was measured at 520 nm using a UV–Vis spectrophotometer. Proline concentration was calculated from a standard curve and expressed as µmol g⁻¹ FW, following Bates et al.^39^.

For pigment analysis, 0.25 g of fresh leaf tissue was extracted in 10 mL absolute ethanol (100%) until the tissue became pale. The absorbance of the extract was measured at 663, 645, and 470 nm using a UV–Vis spectrophotometer, and chlorophyll a (Chl a), chlorophyll b (Chl b), and total chlorophyll (Total Chl) were calculated according to Arnon^40^. Because gas exchange and chlorophyll fluorescence were not measured, these variables are presented as pigment-related traits rather than direct measurements of photosynthetic rate or electron transport.

### Determination of oxidative stress indicators

Oxidative stress was assessed by measuring malondialdehyde (MDA), hydrogen peroxide (H₂O₂), and electrolyte leakage (EL) in leaf tissues. MDA content was estimated according to Heath and Packer^41^. Fresh leaf tissue (0.5 g) was homogenized in 3 mL of 5% trichloroacetic acid (TCA) and centrifuged at "11,500 ×g" for 12 min at 4 °C. The supernatant was mixed with thiobarbituric acid reagent (20% TCA containing 0.5% TBA), heated at 95 °C for 30 min, cooled rapidly, centrifuged again, and absorbance was read at 532 and 600 nm. MDA content was calculated using an extinction coefficient of 155 mM⁻¹ cm⁻¹ and expressed as nmol g⁻¹ FW.

H₂O₂ content was determined following Yu et al.^42^. Fresh leaf tissue (0.5 g) was homogenized in 3 mL of 5% TCA and centrifuged, and 1 mL of the supernatant was mixed with 1 mL of 10 mM potassium phosphate buffer (pH 7.0) and 1 mL of 1 mM potassium iodide. After incubation for 1 h, absorbance was measured at 390 nm and H₂O₂ content was expressed as nmol g⁻¹ FW.

EL was measured according to Dionisio-Sese and Tobita^43^. Leaf tissue (0.5 g) was cut into small pieces and immersed in 15 mL distilled water, then incubated at 40 °C for 60 min. Initial electrical conductivity (EC₁) was measured, after which the samples were autoclaved at 121 °C for 30 min and cooled to room temperature to obtain final electrical conductivity (EC₂). EL was calculated as:

$${\text{EL }}\left( \% \right){\text{ }} = {\text{ }}\left( {{\mathrm{EC}}_{{\mathrm{1}}} {\text{ }}/{\text{ EC}}_{{\mathrm{2}}} } \right){\text{ }} \times {\text{ 1}}00$$

### Quantification of ascorbic acid content

Total ascorbic acid (AsA) was determined following Olgun et al.^44^. Fresh leaf tissue (0.5 g) was homogenized in 3 mL of 5% TCA and centrifuged at 11,500 ×g for 12 min at 4 °C. An aliquot of 0.2 mL supernatant was mixed with 1.8 mL distilled water, followed by the addition of 0.2 mL Folin–Ciocalteu reagent. The mixture was vortexed and incubated for 10 min at 4° C. A blank was prepared in the same way, except that 0.2 mL of 5% TCA replaced the sample extract. Absorbance was recorded at 760 nm, and AsA content was determined according to the corresponding standard procedure and expressed on a fresh-weight basis.

### Statistical analysis

All collected data were subjected to statistical analysis using R software (version 4.5.1). The data were first checked for normality and homogeneity of variances using the Shapiro-Wilk test and Levene’s test, respectively. A two-way factorial ANOVA was performed for each measured trait, with NPK level and biostimulant treatment as fixed factors. The main effects and their interaction were evaluated. When ANOVA indicated significant differences (*p* ≤ 0.05), treatment means were separated using Tukey’s honestly significant difference (HSD) test. Data are presented as mean ± standard error (SE) based on five replicates. Graphical representations were generated using the ggplot2 package in R for visualization of treatment effects. Correlation analysis was carried out to examine the relationships between key physiological and biochemical parameters using the corrplot and performance analytics packages. Principal Component Analysis (PCA) was conducted to identify the major contributing variables and to visualize the treatment clustering patterns based on multidimensional trait data.

## Results

### Vegetative growth and biomass

**Fig. 1:**
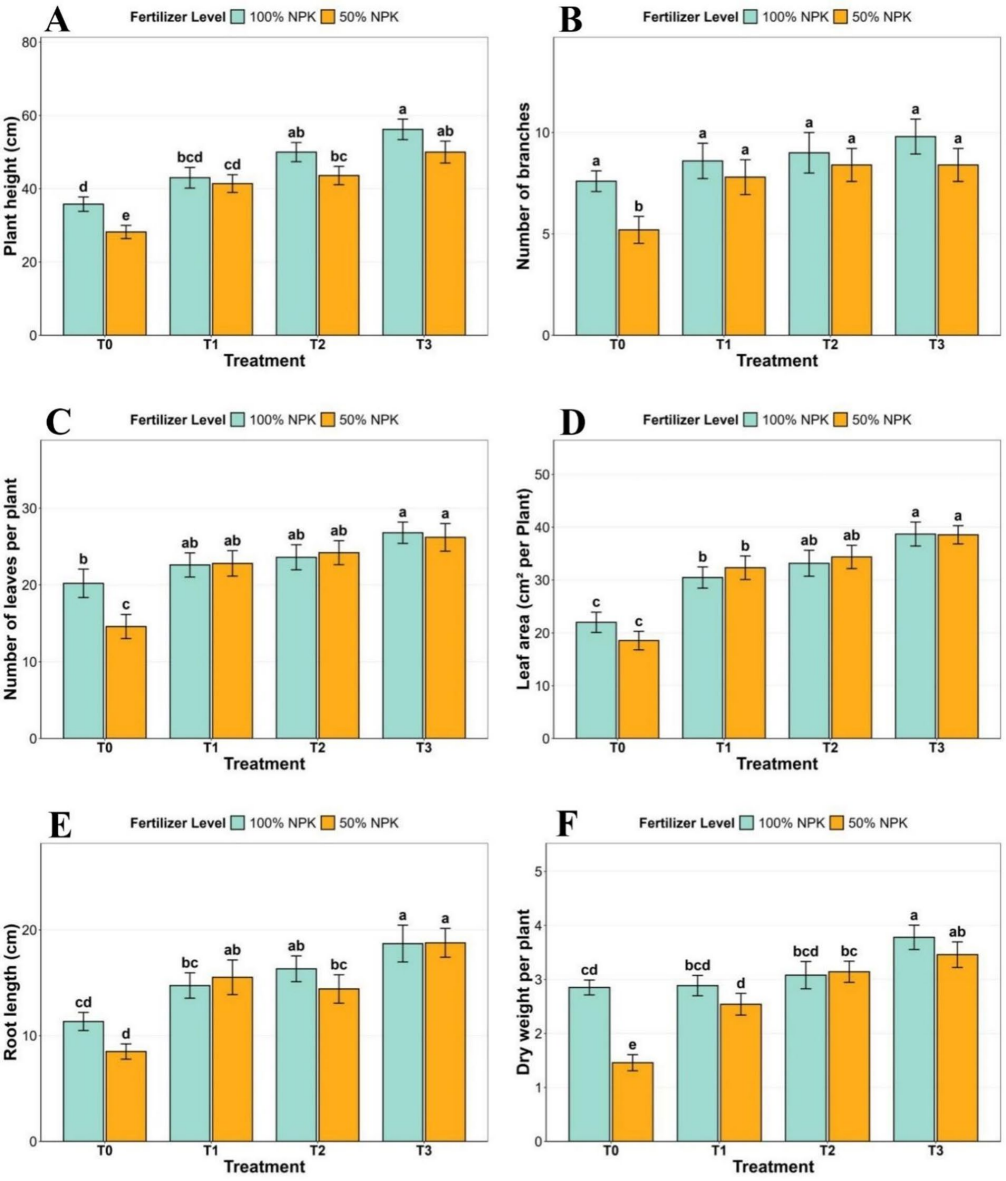
Vegetative growth and biomass of mungbean under four biostimulant treatments (T0–T3) and two fertilizer levels (NPK100 and NPK50). (**A**) Plant height (cm); (**B**) Branch number; (**C**) Leaf number; (**D**) Leaf area (cm² plant^− 1^); (**E**) Root length (cm); (**F**) Dry weight (g plant^− 1^). Values are presented as mean ± SE (*n* = 5); Means not sharing a letter differ significantly according to Tukey’s HSD test at *p* ≤ 0.05. Treatments: T0 = Biochar only; T1 = Biochar + Humic acid; T2 = Biochar + B. amyloliquefaciens; T3 = Biochar + P. fluorescens. Fertilizer levels: NPK100 (100% recommended rate of NPK) and NPK50 (50% recommended rate of NPK).

Vegetative growth and biomass traits differed among treatments, with treatment responses generally more pronounced under NPK50 than under NPK100 (Fig. 1A–F). Under NPK100, T3 showed the most consistent increases relative to T0, with significantly higher plant height, leaf number, root length, and dry biomass. T2 also increased plant height and leaf area compared with T0. Under NPK50, the supplemented treatments (T1–T3) generally exceeded T0, particularly for plant height, leaf number, root length, and dry biomass. Branch number did not differ significantly among treatments under NPK100, whereas both PGPR treatments (T2 and T3) were higher than T0 under NPK50. Leaf area was significantly greater in T2 and T3 than in T0 at both fertilizer levels, while T1 increased leaf area only under NPK50. Overall, T3 showed the most consistent positive response across vegetative and biomass traits. Two-way ANOVA showed a significant NPK × biostimulant interaction for dry weight (*p* = 0.0072), indicating that treatment effects on biomass accumulation differed between NPK levels (Table S1).

### Nodulation and grain yield formation

**Fig. 2:**
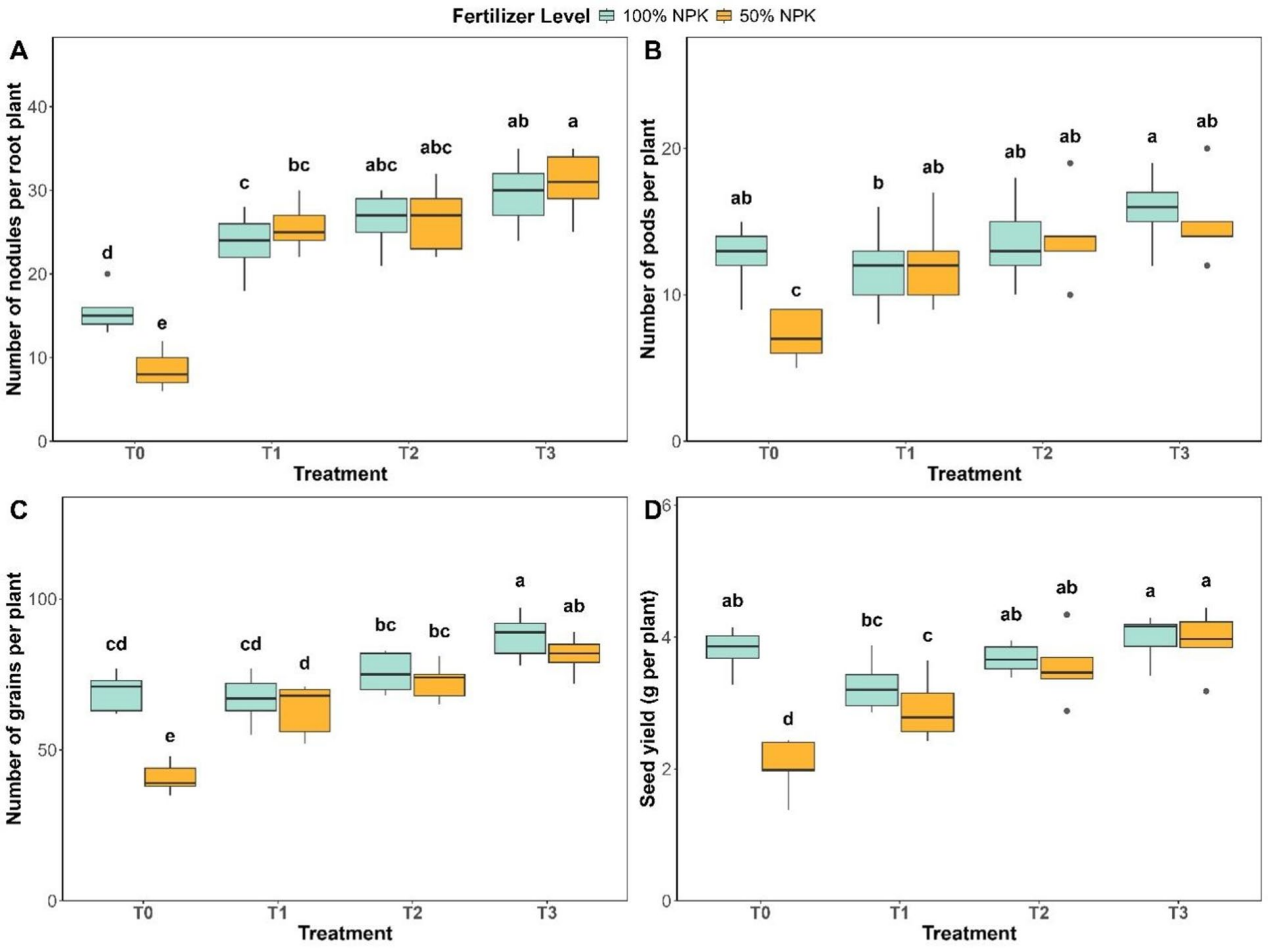
Nodulation and grain yield formation of mungbean under four biostimulant treatments (T0–T3) and two fertilizer levels (NPK100 and NPK50). (**A**) Number of nodules per root plant; (**B**) Number of pods per plant; (**C**) Number of grains per plant; (**D**) Seed yield (g plant^− 1^). Values are presented as mean ± SE (*n* = 5); Means not sharing a letter differ significantly according to Tukey’s HSD test at *p* ≤ 0.05. Treatments: T0 = Biochar only; T1 = Biochar + Humic acid; T2 = Biochar + B. amyloliquefaciens; T3 = Biochar + P. fluorescens. Fertilizer levels: NPK100 (100% recommended rate of NPK) and NPK50 (50% recommended rate of NPK).

Biostimulant supplementation affected nodulation and yield-related traits under both fertilization regimes (Fig. 2A–D). Nodule number increased in all supplemented treatments (T1–T3) compared with the biochar-only control (T0) at both NPK levels, with no significant differences among supplemented treatments (Fig. 2A). Two-way ANOVA showed a significant NPK × biostimulant interaction for nodule number (*p* = 0.0308), indicating that treatment effects differed between NPK levels (Table S1). Pod number did not differ among treatments under NPK100, whereas PGPR treatments increased pod number under NPK50 (Fig. 2B). Grain number increased with biostimulant supplementation at both NPK levels, with T3 showing the highest values overall (Fig. 2C). Significant NPK × biostimulant interactions were observed for grain number (*p* = 0.00068) and grain weight per plant (*p* = 0.00015), indicating fertilizer-level-dependent treatment responses (Table S1). Seed yield followed a similar pattern: under NPK100, both PGPR treatments exceeded T0, while under NPK50 all supplemented treatments exceeded T0 and T3 remained higher than T1 (Fig. 2D). Grain yield under NPK50–T3 was approximately 4.00 g plant⁻¹, compared with approximately 3.85 g plant⁻¹ under NPK100–T0, representing a 3.9% increase relative to the full-NPK biochar-only control. Thus, NPK50–T3 maintained grain yield at a level statistically comparable to NPK100–T0 under the present pot-experimental conditions.

### Photosynthetic pigments and leaf functional status

**Fig. 3:**
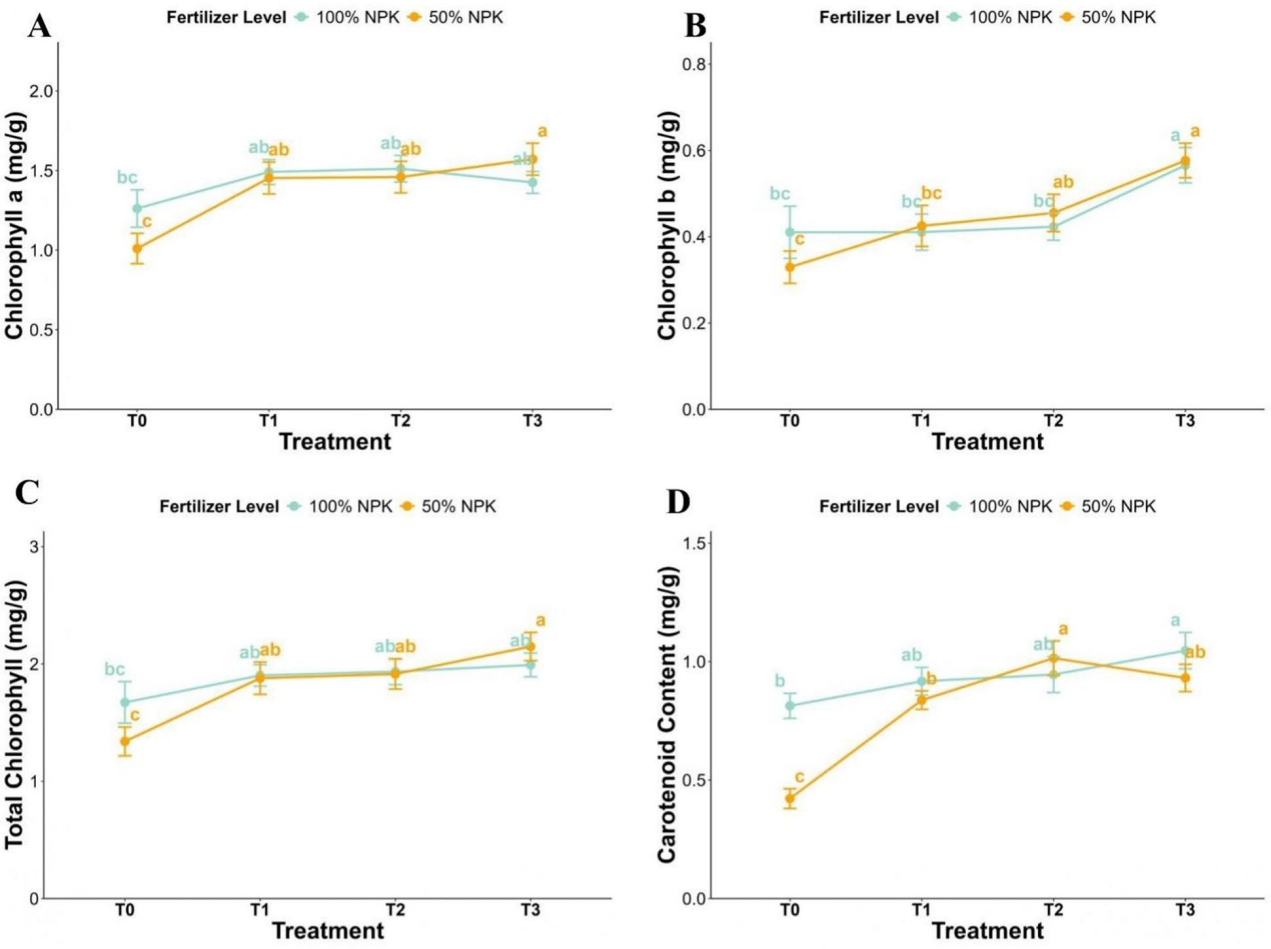
Photosynthetic pigment traits of mungbean under four biostimulant treatments (T0–T3) and two fertilizer levels (NPK100 and NPK50). (**A**) Chl a (mg g⁻¹); (**B**) Chl b (mg g⁻¹); (**C**) Total Chl (mg g⁻¹); and (**D**) Carotenoid content (mg g⁻¹). Values are presented as mean ± SE (n = 5). Means not sharing a letter differ significantly according to Tukey’s HSD test at *p* ≤ 0.05. Treatments: T0 = Biochar only; T1 = Biochar + Humic acid; T2 = Biochar + B. amyloliquefaciens; T3 = Biochar + P. fluorescens. Fertilizer levels: NPK100 (100% recommended rate of NPK) and NPK50 (50% recommended rate of NPK).

Photosynthetic pigment traits responded mainly under reduced fertilization (Fig. 3A–D). Chl a did not differ among treatments under NPK100, whereas all supplemented treatments (T1–T3) exceeded T0 under NPK50 (Fig. 3A). Chl b showed a similar pattern, although only T3 was significantly higher than T0 under NPK50 (Fig. 3B). Total Chl and carotenoid contents were also significantly increased by T1–T3 under NPK50, with no significant treatment effects under NPK100 (Fig. 3C, D). The NPK × biostimulant interaction was significant for carotenoid content (*p* = 0.0060), indicating that treatment effects on carotenoid accumulation differed between NPK levels (Table S1).

### Plant water status and osmotic adjustment

**Fig. 4:**
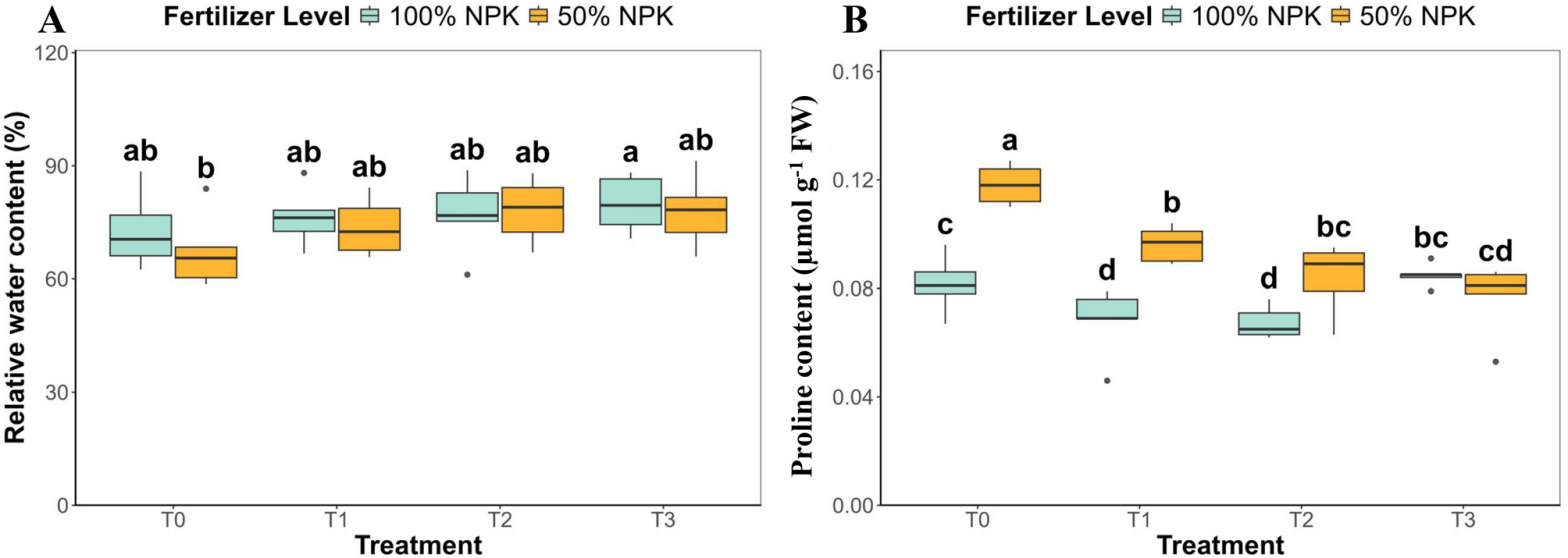
Water status and osmotic adjustment of mungbean under four biostimulant treatments (T0–T3) and two fertilizer levels (NPK100 and NPK50). (**A**) Relative water content; (**B**) Proline content. Values are presented as mean ± SE (n = 5). Means not sharing a letter differ significantly according to Tukey’s HSD test at *p* ≤ 0.05. Treatments: T0 = Biochar only; T1 = Biochar + Humic acid; T2 = Biochar + B. amyloliquefaciens; T3 = Biochar + P. fluorescens. Fertilizer levels: NPK100 (100% recommended rate of NPK) and NPK50 (50% recommended rate of NPK).

Treatment effects on plant water status and osmotic adjustment differed between traits (Fig. 4A, B). Relative water content (RWC) did not differ significantly among treatments under either NPK level (Fig. 4A). In contrast, proline content differed significantly among treatments, particularly under NPK50 (Fig. 4B). Under NPK50, T0 showed higher proline content than T1–T3, with T3 also lower than T1. Under NPK100, T3 showed lower proline content than T1 and T2, while T0 did not differ significantly from the supplemented treatments. Two-way ANOVA showed a significant NPK × biostimulant interaction for proline content (*p* = 0.00011), indicating that treatment effects on proline accumulation differed between NPK levels (Table S1).

### Oxidative stress, membrane stability, and antioxidant defense

**Fig. 5:**
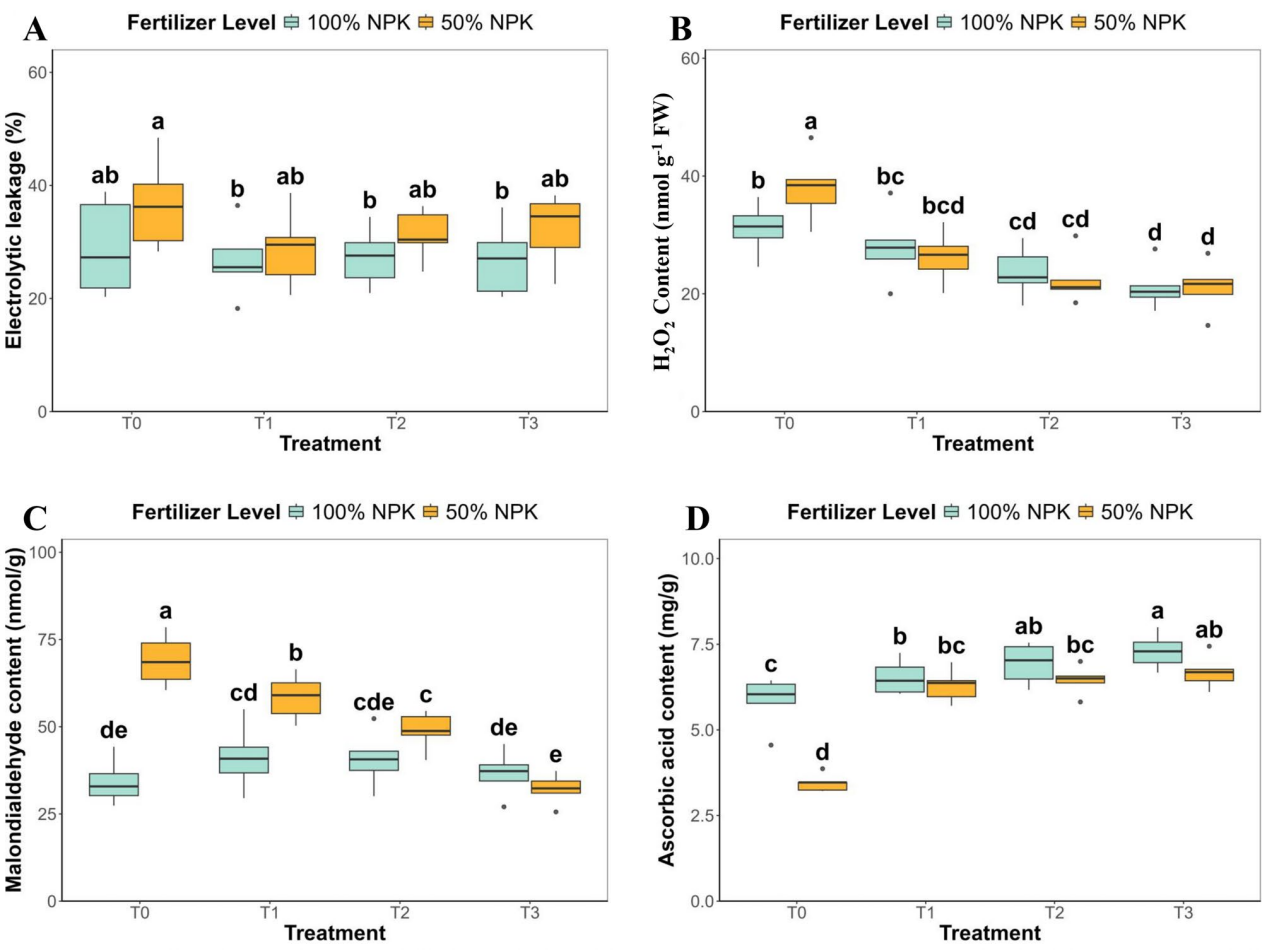
Oxidative stress, membrane stability, and antioxidant defense of mungbean under four biostimulant treatments (T0–T3) and two fertilizer levels (NPK100 and NPK50). (**A**) Electrolytic leakage; (**B**) Hydrogen peroxide content; (**C**) Malondialdehyde content; (**D**) Ascorbic acid content. Values are presented as mean ± SE (n = 5). Means not sharing a letter differ significantly according to Tukey’s HSD test at *p* ≤ 0.05. Treatments: T0 = Biochar only; T1 = Biochar + Humic acid; T2 = Biochar + B. amyloliquefaciens; T3 = Biochar + P. fluorescens. Fertilizer levels: NPK100 (100% recommended rate of NPK) and NPK50 (50% recommended rate of NPK).

Oxidative stress indicators and antioxidant status varied among treatments, with the clearest responses observed under NPK50 (Fig. 5A–D). Electrolyte leakage did not differ significantly among treatments at either NPK level (Fig. 5A). H₂O₂ content declined with biostimulant supplementation, particularly under NPK50; under NPK100, only T3 was lower than T0, whereas under NPK50 all supplemented treatments showed lower H₂O₂ content than T0, with the lowest values in T2 and T3 (Fig. 5B). MDA content showed no significant treatment effect under NPK100 but decreased under NPK50, where T2 and T3 were lower than T0 and T3 was also lower than T1 and T2 (Fig. 5C). Two-way ANOVA showed a significant NPK × biostimulant interaction for MDA content (*p* = 5.04 × 10⁻⁶), indicating that treatment effects on MDA accumulation differed between NPK levels (Table S1). Ascorbic acid content increased with biostimulant supplementation; T2 and T3 exceeded T0 under NPK100, while all supplemented treatments exceeded T0 under NPK50, with T3 showing the highest value (Fig. 5D). 

### Correlation among the key variables influenced by treatments and fertilizer levels

**Fig. 6:**
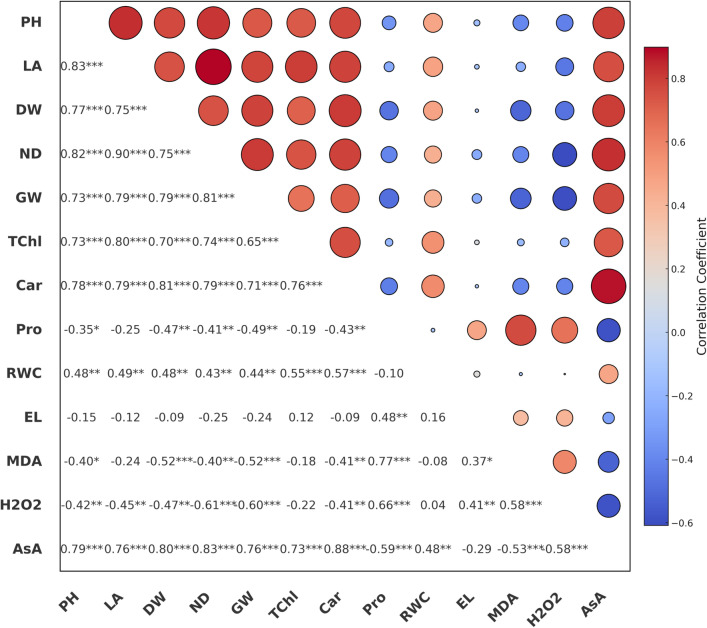
Pearson correlation matrix of physiological, biochemical, and yield-related traits in mungbean under biochar–biostimulant combinations and two NPK fertilization regimes. Asterisks indicate significance levels (*p < 0.05, **p < 0.01, ***p < 0.001). PH: Plant height; LA: Leaf area; DW: Dry weight; ND: Nodule number; GW: Grain weight; TChl: Total Chl; Car: Carotenoids; Pro: Proline; RWC: Relative water content; EL: Electrolyte leakage; MDA: Malondialdehyde; H₂O₂: Hydrogen peroxide; AsA: Ascorbic acid.

Pearson correlation analysis showed consistent associations among growth, physiological, biochemical, and yield-related traits (Fig. 6). Plant height, leaf area, dry weight, nodule number, and grain weight were positively correlated with one another. Total chlorophyll and carotenoid contents were positively associated with nodulation, biomass, and grain weight. In contrast, H₂O₂ and MDA were negatively correlated with growth, pigment content, nodulation, and yield-related traits. Ascorbic acid was positively correlated with pigment content, nodulation, biomass, and grain weight, but negatively correlated with H₂O₂ and MDA.

### Principal component analysis: comprehensive multivariate assessment

**Fig. 7:**
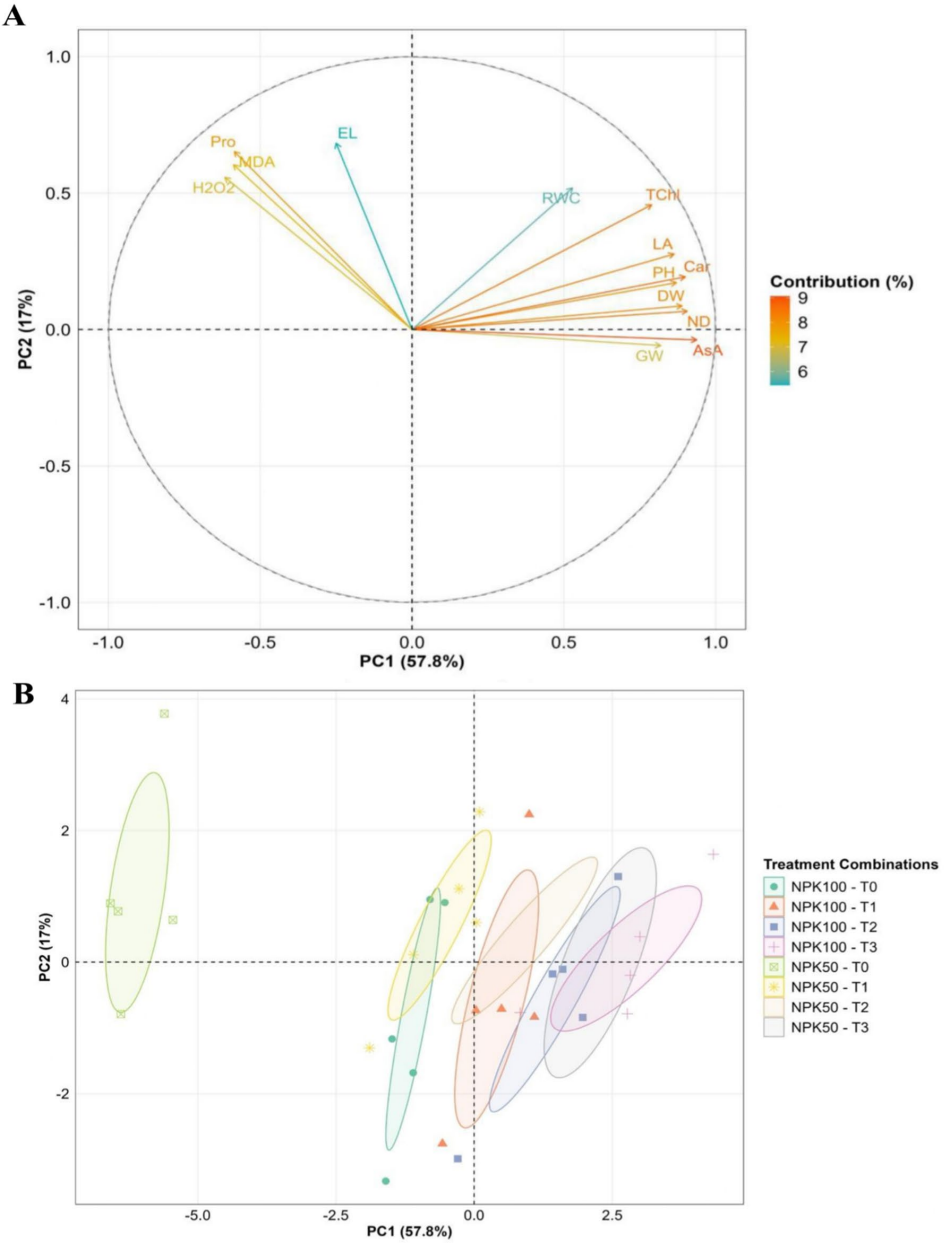
Principal component analysis of growth, physiological, biochemical, and yield-related traits of mungbean under four biostimulant treatments (T0–T3) and two fertilizer levels (NPK100 and NPK50). (**A**) PCA loading plot shows the contribution of individual variables to PC1 and PC2. (**B**) PCA score plot showing the distribution of treatment combinations. Trait abbreviations: PH (plant height), LA (leaf area), DW (dry weight), GW (grain weight), Car (carotenoids), TChl (total chlorophyll), RWC (relative water content), EL (electrolyte leakage), H₂O₂ (hydrogen peroxide), MDA (malondialdehyde), Pro (proline), AsA (ascorbic acid), and ND (nodule number). T0 = biochar only, T1 = biochar + humic acid, T2 = biochar + Bacillus amyloliquefaciens, and T3 = biochar + Pseudomonas fluorescens. Fertilizer levels were NPK100 = 100% recommended NPK dose and NPK50 = 50% recommended NPK dose.

Principal component analysis (PCA) summarized the multivariate treatment responses effectively, with PC1 and PC2 explaining 57.8% and 17.0% of the total variance, respectively (74.8% combined) (Fig. 7A, B). Positive loadings on PC1 were associated mainly with ascorbic acid, chlorophyll, carotenoids, plant height, leaf area, dry weight, nodule number, and grain weight, whereas proline, H₂O₂, and MDA loaded in the opposite direction (Fig. 7A, Table S2). PC2 was influenced primarily by relative water content and electrolyte leakage. The score plot showed clear separation among treatment combinations, particularly between NPK50–T0 and the supplemented treatments (Fig. 7B). The NPK50–T0 group was positioned apart from the other treatments and aligned more closely with stress-related traits, whereas T1–T3 were distributed toward the positive side of PC1 together with growth-, pigment-, antioxidant-, and yield-related variables. Among the reduced-fertilizer treatments, NPK50–T3 clustered closest to the NPK100 treatment groups.

## Discussion

### Biostimulant supplementation was most effective under reduced NPK in a biochar-amended system

The present study showed that biostimulant supplementation under a common biochar-amended background produced clearer responses under 50% NPK than under full fertilization. Across vegetative growth, nodulation, pigment traits, antioxidant status, and yield-related variables, treatment differences were generally larger and more consistent under NPK50, whereas responses under NPK100 were smaller or trait-specific. This pattern is consistent with the broader view that biostimulants often exert stronger effects under nutritional or physiological limitation than under non-limiting conditions^17,45^. A key contribution of this study is that humic acid, *Bacillus amyloliquefaciens*, and *Pseudomonas fluorescens* were compared within the same biochar-amended system under contrasting NPK levels, allowing treatment-specific biostimulant responses to be evaluated under reduced fertilization.

Because biochar was included in all treatments, the observed responses should be interpreted as the effects of humic acid or PGPR supplementation relative to a shared biochar-amended control, rather than as independent effects of biochar itself. Within this framework, supplemented treatments were associated with improved mungbean performance under reduced fertilization, plausibly reflecting complementary contributions of biochar-associated nutrient retention and biostimulant-mediated root or microbial activity^23,46–54^. However, these processes were not directly quantified in the present study; therefore, they are considered plausible explanations rather than demonstrated mechanisms.

### Nodulation and yield responses indicate improved performance under reduced fertilization

Nodulation was one of the most responsive traits and was positively associated with biomass and grain yield. All supplemented treatments increased nodule number relative to the biochar-only treatment at both fertilizer levels, and this response was accompanied by improved reproductive performance, especially under NPK50. Nodulation is closely linked to plant nitrogen status and influences vegetative development and yield formation in legumes^54–57^. The positive relationships among nodule number, canopy development, biomass, and grain yield in the present study indicate that improved nodulation was an important component of the overall treatment response under reduced fertilization.

The yield response further supports this interpretation. Although pod number was less responsive than grain number and grain yield, the supplemented treatments generally improved reproductive output, with the clearest response under NPK50. Notably, NPK50–T3 maintained grain yield at a level comparable to NPK100–T0 under the present pot-experimental conditions, indicating that *P. fluorescens* supplementation helped sustain yield performance despite reduced NPK input. This finding is agronomically significant because it suggests that improved nodulation and physiological performance may contribute to sustaining mungbean yield under reduced mineral fertilizer input, with relevance to lower-input nutrient management in grain legume systems. However, increased nodulation should be interpreted as a treatment-associated response rather than direct evidence of specific microbial interaction or enhanced biological nitrogen fixation, as these mechanisms were not directly measured. Even so, improved nodulation may have contributed to better nitrogen status and greater resilience of mungbean productivity in the low-input system^58–60^. Nevertheless, this interpretation remains conditional on the experimental scale, and field validation is required before recommending fertilizer reduction under practical production conditions.

In the present study, both PGPR treatments increased nodulation, biomass, and grain yield relative to the biochar-only treatment, consistent with reports that biochar can improve the rhizosphere environment in legumes and that PGPR can complement this through nutrient mobilization and plant growth-promoting activity^61–66^. However, rhizosphere colonization, strain persistence, and direct effects on symbiotic efficiency were not measured during the experiment. Therefore, the present findings support an association between PGPR supplementation and improved nodulation-related performance rather than direct confirmation of the specific pathways involved. Humic acid also improved several traits relative to the biochar-only control, although its effects were generally smaller than those of the PGPR treatments. This intermediate response is consistent with previous reports on humic substance activity under suboptimal conditions^67^. The contrast between humic acid and PGPR treatments suggests that microbial inoculants may provide broader functional benefits than humic inputs alone under reduced fertilization.

### Pigment maintenance and antioxidant regulation were associated with improved plant performance

Biostimulant supplementation, especially in the PGPR-amended treatments, was associated with higher chlorophyll and carotenoid contents under NPK50. These pigment responses were positively related to biomass and grain yield, consistent with more favorable leaf nutritional and metabolic status and with a possible photoprotective role of carotenoids under stress conditions^68–70^. However, because gas exchange, chlorophyll fluorescence, and electron transport were not measured, these results should be interpreted as improved pigment status rather than direct evidence of enhanced photosynthetic efficiency. Even so, the positive associations among pigment traits, nodulation, biomass, and grain yield suggest that maintenance of leaf pigment pools was linked with improved plant performance under low nutrient input^71,72^.

The biochemical responses were consistent with this pattern. Under NPK50, supplemented treatments generally reduced H₂O₂ and MDA while increasing ascorbic acid. These responses were accompanied by improved growth and yield-related traits, and the correlation analysis showed negative associations of H₂O₂ and MDA with nodulation, pigment content, biomass, and grain yield. In contrast, ascorbic acid was positively associated with these agronomic and physiological variables. These relationships indicate that lower oxidative burden and stronger antioxidant status were closely linked with improved treatment performance under reduced fertilization.

Relative water content showed comparatively small variation among treatments, suggesting that biostimulant-induced improvements in plant performance under reduced NPK were not primarily mediated by bulk leaf water retention. By contrast, proline, H₂O₂, and MDA were more responsive, particularly under reduced fertilization. Electrolyte leakage also varied less consistently than the biochemical stress indicators. This pattern suggests that treatment effects were expressed more strongly through metabolic and oxidative-stress markers than through large differences in bulk tissue hydration. Similar responses have been reported in other nutrient- and stress-related studies, where biochemical indicators responded more sensitively than leaf water status alone^50,51,73^.

Ascorbic acid is an important antioxidant, whereas H₂O₂, and MDA are established oxidative-stress markers^16,74–77^. In the present study, the inverse relationship between oxidative markers and agronomic traits, together with the positive association of AsA with pigments, biomass, and grain yield, indicates that antioxidant buffering was closely associated with the treatment response. Because antioxidant enzymes and redox-related signaling pathways were not directly measured, these relationships should be interpreted as biologically plausible associations rather than direct evidence of specific biochemical pathways.

### Multivariate analysis integrated the growth, physiological, and stress-response patterns

The multivariate analyses supported the patterns observed in the univariate results. Correlation analysis showed that growth, nodulation, pigment content, and grain yield were positively associated with one another, whereas oxidative stress indicators were negatively associated with these traits. Principal component analysis further separated the NPK50–T0 treatment from the supplemented treatments, with the latter aligning more closely with positive agronomic and physiological variables. Among the reduced-fertilizer treatments, NPK50–T3 clustered closest to the NPK100 groups, indicating that its overall trait profile was more similar to that of the higher-fertility treatments than to the low-input control. This convergence across multivariate and univariate analyses supports the interpretation that the treatment response reflected coordinated changes across growth, nodulation, pigment status, oxidative-stress markers, antioxidant status, biomass, and yield-related traits rather than isolated trait-level effects. Among the supplemented treatments, *P. fluorescens* (T3) produced the most consistent positive response, particularly under NPK50. *Bacillus amyloliquefaciens* (T2) also improved several traits relative to the biochar-only control, whereas humic acid generally produced intermediate responses. This treatment-specific pattern suggests that biostimulant identity influenced plant performance under reduced fertilization.

### Agronomic relevance and study limitations

Overall, the results indicate that supplementing biochar with humic acid or PGPR was associated with improved mungbean performance under reduced mineral fertilization. Across the measured traits, the response pattern was coherent: supplemented treatments showed higher nodulation, improved pigment status, lower oxidative stress, stronger antioxidant capacity, and better biomass and grain yield than the biochar-only control, with the clearest effects under NPK50. This coordinated response highlights the broader significance of biochar-assisted biostimulant supplementation as a potential lower-input nutrient-management approach for grain legumes. These findings support the potential value of microbial biostimulant supplementation within biochar-amended low-input legume systems, while recognizing that the study was not designed to isolate biochar effects independently.

Several limitations should nevertheless be acknowledged. The study was conducted under controlled pot and polytunnel conditions, which are useful for screening treatment effects but may not fully represent field-scale variation in soil structure, root exploration, weather, and microbial competition. Although the PGPR strains were characterized before application using standard biochemical and plant growth-promoting trait assays, rhizosphere colonization, persistence, and population dynamics were not quantified during the experiment. Therefore, while viable strains were applied at known CFU concentrations, their establishment on roots, survival over time, and competitiveness with native microorganisms could not be confirmed. This precludes direct attribution of the observed agronomic and physiological benefits to in situ PGPR activity rather than to other treatment-associated effects. Post-harvest soil chemical and biological properties were also not measured, so nutrient-retention effects, residual biochar effects, and microbial changes remain inferential.

The 5% (v/v) biochar rate used in this pot experiment requires careful agronomic interpretation. Based on the measured biochar bulk density of 0.28 kg L⁻¹, this rate is approximately equivalent to 14 t ha⁻¹ when shallow incorporation into the upper 10 cm of soil is assumed. The estimated field-equivalent biochar rate of 14 t ha⁻¹ falls within the range used in some field applications. For example, Pei et al. applied 10–20 t ha⁻¹ yr⁻¹ biochar in a five-year field experiment under reduced fertilization and irrigation^78^. However, its economic feasibility remains context-dependent because profitability depends on biochar cost, feedstock availability, transport distance, application method, crop response, persistence of benefits, and potential carbon-credit support^79–81^.

Despite these limitations, the present findings provide consistent evidence that biochar-based supplementation, particularly with *Pseudomonas fluorescens*, can help maintain mungbean productivity under reduced mineral NPK input. The grain yield achieved with *P. fluorescens* at 50% NPK, when expressed as an approximate pot-to-area equivalent, was about 1.85 t ha⁻¹. This value is within the range of published mungbean yields reported under full NPK management in comparable production contexts, including reported yields of approximately 1.6–1.8 t ha⁻¹ for BARI Mung-6^35^. However, because this estimate was derived from a controlled pot experiment, it should be interpreted cautiously and validated under field conditions. Future work should include multi-location and multi-season field trials, post-harvest soil analyses, microbial profiling, strain-colonization assays, antioxidant enzyme measurements, gas-exchange analysis, and isotope-based approaches such as ¹⁵N tracing to clarify mechanisms and assess agronomic and economic scalability.

## Conclusion

In conclusion, biostimulant supplementation within a common biochar-amended system improved mungbean performance under reduced mineral fertilization, with the clearest responses observed at 50% NPK. Across growth, nodule number, pigment status, oxidative-stress indicators, antioxidant capacity, and yield-related traits, the supplemented treatments generally outperformed the biochar-only control. Among the tested amendments, *Pseudomonas fluorescens* produced the most consistent positive response, and NPK50–T3 reached a grain yield of 4.00 g plant⁻¹, statistically comparable to the 3.85 g plant⁻¹ recorded under the full-NPK biochar-only control under the present pot-experimental conditions. This treatment also enhanced nodulation, increased total chlorophyll by approximately 60%, and reduced malondialdehyde content by approximately 50%, suggesting improved photosynthetic and oxidative-stress status under reduced fertilizer input. These findings identify biochar-assisted *P. fluorescens* supplementation as a promising strategy for sustaining mungbean productivity under reduced NPK fertilization. However, these findings should be interpreted within the scope of the controlled pot and polytunnel experiment, as rhizosphere colonization, post-harvest soil responses, gas exchange, biological nitrogen fixation and molecular mechanisms were not directly assessed. Field-scale validation, ¹⁵N tracing, and rhizosphere microbial profiling are therefore needed to confirm the agronomic scalability and mechanistic basis of these responses.

## Supplementary Information

Below is the link to the electronic supplementary material.

Supplementary Material 1

## Data Availability

The datasets generated during and/or analysed during the current study are available from the corresponding author on reasonable request.
