# Supplementary material for: Biochar-biostimulant combinations enhance nodulation, yield, and antioxidant defense in mungbean under reduced NPK fertilization
Source: Sci Rep. 2026 Jul 30;16:23614. doi: 10.1038/s41598-026-63172-0 (PMC13424373; doi:10.1038/s41598-026-63172-0)
Supplement: Supplementary file 1 — Supplementary Material 1 [file 41598_2026_63172_MOESM1_ESM.docx]

**Biochar-biostimulant combinations enhance nodulation, yield, and antioxidant defense in mungbean under reduced NPK fertilization**

**Table S1.** Two‑way ANOVA F‑values, p‑values, and significance of NPK × biostimulant interaction for all measured traits. Factor.A= NPK level, Factor.B= Biostimulant treatment

| Response | Source | df | SS | MS | F | p_value |
| --- | --- | --- | --- | --- | --- | --- |
| Plant Height | Factor.A | 1 | 297.025 | 297.025 | 9.347757671 | 0.004483948 |
|  | Factor.B | 3 | 2369.875 | 789.9583333 | 24.86100184 | 1.70506E-08 |
|  | Factor.A:Factor.B | 3 | 52.275 | 17.425 | 0.548387097 | 0.652853078 |
|  | Residuals | 32 | 1016.8 | 31.775 |  |  |
| No of Branch | Factor.A | 1 | 16.9 | 16.9 | 5.140684411 | 0.03026295 |
|  | Factor.B | 3 | 42.6 | 14.2 | 4.319391635 | 0.011496108 |
|  | Factor.A:Factor.B | 3 | 4.9 | 1.633333333 | 0.496831432 | 0.687077234 |
|  | Residuals | 32 | 105.2 | 3.2875 |  |  |
| No of Leaf Plant | Factor.A | 1 | 18.225 | 18.225 | 1.363891487 | 0.251492246 |
|  | Factor.B | 3 | 439.475 | 146.4916667 | 10.96289367 | 4.13591E-05 |
|  | Factor.A:Factor.B | 3 | 62.075 | 20.69166667 | 1.548487683 | 0.221027532 |
|  | Residuals | 32 | 427.6 | 13.3625 |  |  |
| Leaf Area Plant | Factor.A | 1 | 0.18225 | 0.18225 | 0.008365416 | 0.927695149 |
|  | Factor.B | 3 | 1806.89475 | 602.29825 | 27.64595585 | 5.16977E-09 |
|  | Factor.A:Factor.B | 3 | 42.04475 | 14.01491667 | 0.643295523 | 0.592846712 |
|  | Residuals | 32 | 697.156 | 21.786125 |  |  |
| Root Length | Factor.A | 1 | 9.409 | 9.409 | 1.111042229 | 0.299749554 |
|  | Factor.B | 3 | 397.714 | 132.5713333 | 15.65441064 | 1.92184E-06 |
|  | Factor.A:Factor.B | 3 | 21.317 | 7.105666667 | 0.839057895 | 0.48252475 |
|  | Residuals | 32 | 270.996 | 8.468625 |  |  |
| DW | Factor.A | 1 | 2.488313689 | 2.488313689 | 12.22789735 | 0.001404662 |
|  | Factor.B | 3 | 11.51383815 | 3.837946048 | 18.86016643 | 3.15645E-07 |
|  | Factor.A:Factor.B | 3 | 2.927530933 | 0.975843644 | 4.795422685 | 0.007186807 |
|  | Residuals | 32 | 6.511834028 | 0.203494813 |  |  |
| No of Nodule in Root Plant | Factor.A | 1 | 8.1 | 8.1 | 0.63654224 | 0.43084674 |
|  | Factor.B | 3 | 1849.7 | 616.5666667 | 48.45317616 | 5.30633E-12 |
|  | Factor.A:Factor.B | 3 | 128.1 | 42.7 | 3.355599214 | 0.030864809 |
|  | Residuals | 32 | 407.2 | 12.725 |  |  |
| No of Pod Plant | Factor.A | 1 | 18.225 | 18.225 | 2.317965024 | 0.137709128 |
|  | Factor.B | 3 | 168.075 | 56.025 | 7.125596184 | 0.000848338 |
|  | Factor.A:Factor.B | 3 | 57.075 | 19.025 | 2.419713831 | 0.08424518 |
|  | Residuals | 32 | 251.6 | 7.8625 |  |  |
| No of Grain Plant | Factor.A | 1 | 1050.625 | 1050.625 | 20.90276051 | 6.86334E-05 |
|  | Factor.B | 3 | 4756.475 | 1585.491667 | 31.54422615 | 1.11471E-09 |
|  | Factor.A:Factor.B | 3 | 1113.275 | 371.0916667 | 7.383072204 | 0.00068034 |
|  | Residuals | 32 | 1608.4 | 50.2625 |  |  |
| Grain Weight per Plant | Factor.A | 1 | 3.27184 | 3.27184 | 18.6862181 | 0.000140306 |
|  | Factor.B | 3 | 6.84611 | 2.282036667 | 13.03322744 | 9.93729E-06 |
|  | Factor.A:Factor.B | 3 | 4.84196 | 1.613986667 | 9.217842822 | 0.000152714 |
|  | Residuals | 32 | 5.603 | 0.17509375 |  |  |
| Chl a | Factor.A | 1 | 0.023961025 | 0.023961025 | 0.542573405 | 0.466734366 |
|  | Factor.B | 3 | 0.919611675 | 0.306537225 | 6.941228352 | 0.000995337 |
|  | Factor.A:Factor.B | 3 | 0.197808275 | 0.065936092 | 1.493056737 | 0.235102434 |
|  | Residuals | 32 | 1.413178 | 0.044161813 |  |  |
| Chl b | Factor.A | 1 | 0.0003481 | 0.0003481 | 0.0366055 | 0.849479289 |
|  | Factor.B | 3 | 0.2223785 | 0.074126167 | 7.794959427 | 0.00048079 |
|  | Factor.A:Factor.B | 3 | 0.0194353 | 0.006478433 | 0.681259092 | 0.570010281 |
|  | Residuals | 32 | 0.304304 | 0.0095095 |  |  |
| Chl a b | Factor.A | 1 | 0.030085225 | 0.030085225 | 0.377688725 | 0.543188205 |
|  | Factor.B | 3 | 1.738515875 | 0.579505292 | 7.275086514 | 0.000746055 |
|  | Factor.A:Factor.B | 3 | 0.310194475 | 0.103398158 | 1.298056391 | 0.292016905 |
|  | Residuals | 32 | 2.5489964 | 0.079656138 |  |  |
| Carotenoid | Factor.A | 1 | 0.167314225 | 0.167314225 | 9.006234659 | 0.005180944 |
|  | Factor.B | 3 | 0.897799475 | 0.299266492 | 16.10899641 | 1.46892E-06 |
|  | Factor.A:Factor.B | 3 | 0.277167075 | 0.092389025 | 4.973141041 | 0.006048969 |
|  | Residuals | 32 | 0.5944832 | 0.0185776 |  |  |
| Proline | Factor.A | 1 | 0.0033489 | 0.0033489 | 33.91720471 | 1.80854E-06 |
|  | Factor.B | 3 | 0.0033705 | 0.0011235 | 11.37865553 | 3.07467E-05 |
|  | Factor.A:Factor.B | 3 | 0.0028569 | 0.0009523 | 9.64476516 | 0.000109872 |
|  | Residuals | 32 | 0.0031596 | 9.87375E-05 |  |  |
| RWC | Factor.A | 1 | 50.40025 | 50.40025 | 0.614216794 | 0.438967202 |
|  | Factor.B | 3 | 446.14475 | 148.7149167 | 1.812356075 | 0.164756928 |
|  | Factor.A:Factor.B | 3 | 56.94875 | 18.98291667 | 0.231340642 | 0.873868348 |
|  | Residuals | 32 | 2625.796 | 82.056125 |  |  |
| Electrolytic Leakage | Factor.A | 1 | 224.3348496 | 224.3348496 | 4.983304539 | 0.032718238 |
|  | Factor.B | 3 | 137.2504454 | 45.75014847 | 1.016279561 | 0.398336095 |
|  | Factor.A:Factor.B | 3 | 42.7076302 | 14.23587673 | 0.316231336 | 0.813520042 |
|  | Residuals | 32 | 1440.553177 | 45.01728679 |  |  |
| MDA | Factor.A | 1 | 1935.298323 | 1935.298323 | 40.08985781 | 4.16983E-07 |
|  | Factor.B | 3 | 1810.062865 | 603.3542883 | 12.4985318 | 1.41942E-05 |
|  | Factor.A:Factor.B | 3 | 2039.892184 | 679.9640614 | 14.08550931 | 5.03605E-06 |
|  | Residuals | 32 | 1544.76842 | 48.27401314 |  |  |
| H_2_O_2_ | Factor.A | 1 | 9.98001 | 9.98001 | 0.431833198 | 0.515789738 |
|  | Factor.B | 3 | 1053.797425 | 351.2658083 | 15.19920694 | 2.52679E-06 |
|  | Factor.A:Factor.B | 3 | 123.7588228 | 41.25294093 | 1.78500717 | 0.169846398 |
|  | Residuals | 32 | 739.5455504 | 23.11079845 |  |  |
| As A | Factor.A | 1 | 8.612768025 | 8.612768025 | 31.66442971 | 3.20312E-06 |
|  | Factor.B | 3 | 33.43330988 | 11.14443663 | 40.97198824 | 4.554E-11 |
|  | Factor.A:Factor.B | 3 | 7.179259275 | 2.393086425 | 8.798067785 | 0.000212469 |
|  | Residuals | 32 | 8.7040436 | 0.272001363 |  |  |

| Variable | Dim.1 | Dim.2 | Dim.3 | Dim.4 | Dim.5 |
| --- | --- | --- | --- | --- | --- |
| PH | 0,87574343 | 0,16838423 | 0,11132318 | 0,02091851 | 0,17054647 |
| LA | 0,87646302 | 0,27146627 | 0,29240864 | -0,0295433 | -0,0406935 |
| DW | 0,8862634 | 0,0862115 | -0,1487517 | -0,1118314 | 0,12223265 |
| ND | 0,9176752 | 0,06189227 | 0,25892505 | -0,0316379 | -0,0791501 |
| GW | 0,88671477 | -0,0407137 | 0,05701168 | -0,0630616 | -0,1722738 |
| TChl | 0,78344417 | 0,45718402 | 0,02641685 | -0,0733466 | 0,14886602 |
| Car | 0,89301732 | 0,19287023 | -0,097107 | 0,04302408 | 0,12156771 |
| Pro | -0,580817 | 0,65249695 | 0,32869317 | -0,036815 | 0,01156604 |
| AsA | 0,93467854 | -0,039775 | -0,0289714 | 0,07693986 | 0,08512129 |
| RWC | 0,52604172 | 0,51950379 | -0,3845556 | 0,42208658 | -0,341709 |
| EL | -0,2543549 | 0,68578597 | -0,3614365 | -0,5591 | -0,0884495 |
| MDA | -0,5719642 | 0,60058626 | 0,409822 | 0,09744912 | -0,1267698 |
| H2O2 | -0,6264576 | 0,56202294 | -0,1894293 | 0,30508897 | 0,33507921 |

Supplementary Table S2. PCA loading values for the first two principal components.
